# Granulocyte‐colony stimulating factor‐producing uterine cervical cancer treated with chemoradiotherapy: A case report with mutation analysis and literature review

**DOI:** 10.1002/ccr3.3495

**Published:** 2020-11-06

**Authors:** Shintaro Shiba, Takahiro Oike, Ken Ando, Yuya Yoshimoto, Yoshiyasu Takayama, Tatsuya Ohno

**Affiliations:** ^1^ Department of Radiation Oncology Gunma University Graduate School of Medicine Maebashi Japan; ^2^ Gunma University Heavy Ion Medical Center Maebashi Japan; ^3^ Department of Radiation Oncology, School of Medicine Fukushima Medical University Hikarigaoka Japan; ^4^ Department of Pathology Gunma University Hospital Maebashi Japan

**Keywords:** granulocyte‐colony stimulating factor, radiotherapy, somatic mutations, uterine cervical cancer

## Abstract

Granulocyte‐colony stimulating factor‐producing uterine cervical cancer is a rare aggressive disease, which may be genetically distinct from other uterine cervical cancers.

## Graphical abstract

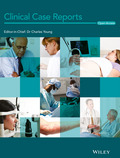

## 1 INTRODUCTION

Granulocyte‐colony stimulating factor (G‐CSF)‐producing uterine cervical cancer (UCC) is a rare aggressive disease. We report a case of G‐CSF‐producing UCC treated with chemoradiotherapy. Target‐capture sequencing results indicate that G‐CSF‐producing UCC is genetically distinct from other UCCs, warranting further research to establish specific treatment strategies.

UCC is a common cancer that causes >311 000 deaths worldwide each year.^1^ Advances in radiotherapy and chemotherapy have markedly improved the treatment outcomes of UCC.^2^ Translational research efforts have provided information on the biological properties of tumors associated with treatment responses in patients with UCC,^3^, ^4^, ^5^ which may help to personalize treatment strategies.

Although UCC is a common cancer, a subset that produces G‐CSF is rare. G‐CSF‐producing UCCs are highly aggressive.^6^, ^7^, ^8^, ^9^, ^10^, ^11^, ^12^, ^13^ However, specific patterns of care for G‐CSF‐producing UCCs have not been established, and treatment outcomes remain unknown because of the limited number of reported cases. Moreover, the mutation profile of G‐CSF‐producing UCC has not been analyzed. Here, we report a case of G‐CSF‐producing UCC, and the results of mutational analysis and literature review.

## 2 CASE REPORT

**FIGURE 1:**
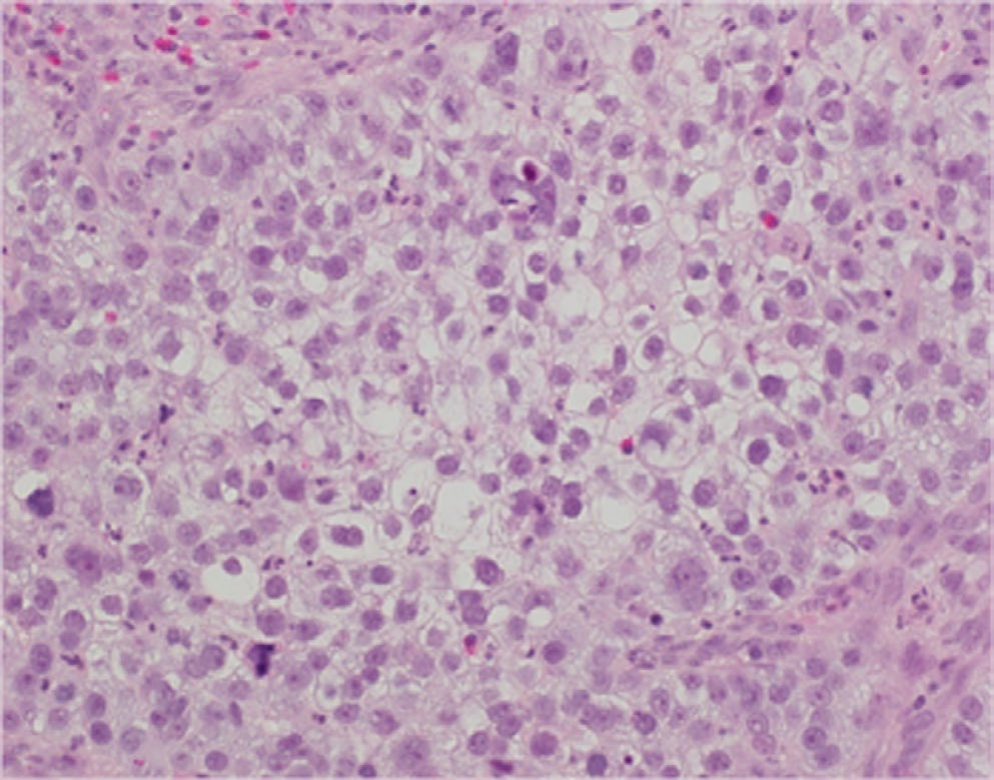
Hematoxylin‐eosin staining of biopsied tumor tissue (×400)

**FIGURE 2:**
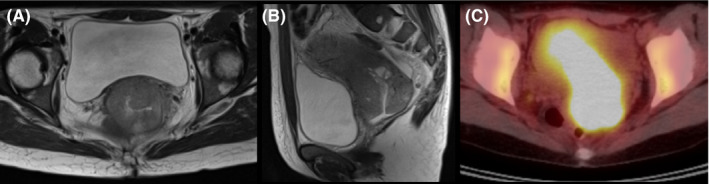
Magnetic resonance imaging (MRI) and ^18^fluoro‐2‐deoxyglucose‐positron emission tomography (FDG‐PET) showing the primary tumor before the initiation of chemoradiotherapy. A, MRI axial plane. B, MRI sagittal plane. C, FDG‐PET axial plane

**FIGURE 3:**
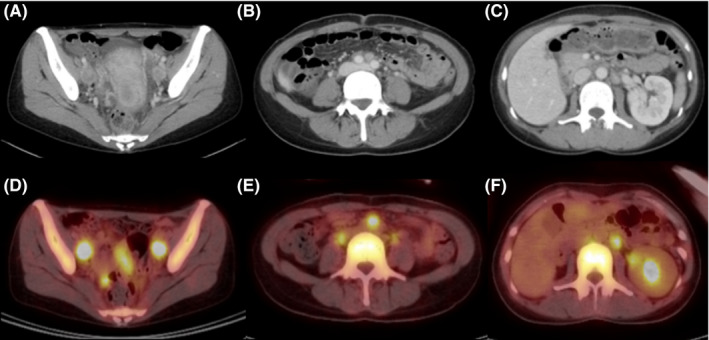
Computed tomography (CT) and ^18^fluoro‐2‐deoxyglucose‐positron emission tomography (FDG‐PET) showing multiple lymphadenopathy before the initiation of chemoradiotherapy. A‐C, CT image showing enlarged lymph nodes in the external iliac, common iliac, and para‐aortic lymph node regions, respectively. D‐F, FDG‐PET images corresponding to A‐C

A 43‐year‐old Japanese woman with UCC was referred to the Department of Radiation Oncology from the Department of Gynecology for definitive radiotherapy. Histological analysis of the biopsy specimen showed the presence of tumor cells with enlarged nuclei and pale‐to‐clear cytoplasm exhibiting sheet‐like growth and stromal infiltration (Figure 1). Immunohistochemical analysis of the biopsy specimen showed positivity for p16, p53, and p63, leading to the diagnosis of squamous cell carcinoma. In line with the pathological diagnosis, the patient showed high levels of serum squamous carcinoma antigen (SCC, 19.0 ng/mL; normal, <1.5 ng/mL). Genotyping of the biopsy specimen showed positivity for human papillomavirus (HPV) type 31. Bimanual pelvic examination revealed bilateral parametrium involvement not reaching the pelvic sidewalls and the absence of vaginal involvement. Magnetic resonance imaging detected a tumor (61 × 60 × 75 mm) located in the uterine cervix with slightly increased intensity on T2‐weighted images extending to the bilateral parametria (Figure 2A,B). Computed tomography (CT) showed lymphadenopathy in the external iliac, common iliac, and para‐aortic lymph node (PALN) regions (Figure 3A‐C). 2‐deoxy‐2‐[^18^F]fluoro‐D‐glucose (FDG)‐positron emission tomography (PET) showed abnormal FDG uptake in the primary tumor (Figure 2C) and in the involved lymph nodes (Figure 3D‐F). The CT and FDG‐PET examinations showed no evidence of metastasis to distant organ sites. The patient was diagnosed as stage IIB based on the 2008 definition by the International Federation of Gynecology and Obstetrics.

The patient had a high‐grade fever (39.1°C) with no symptoms of infection. Blood tests showed a high white blood cell (WBC) count (28.3 × 10^3^/mm^3^; normal, 3.0‐9.0 × 10^3^/mm^3^) and high levels of serum C‐reactive protein (CRP, 6.21 mg/dL; normal, <1.5 mg/dL). Immature granulocytes suggestive of leukemia were not detected in the peripheral blood. Serum G‐CSF was abnormally high (323 pg/mL; normal, <39 pg/mL). Based on these data, the clinical diagnosis was G‐CSF‐producing tumor.

**FIGURE 4:**
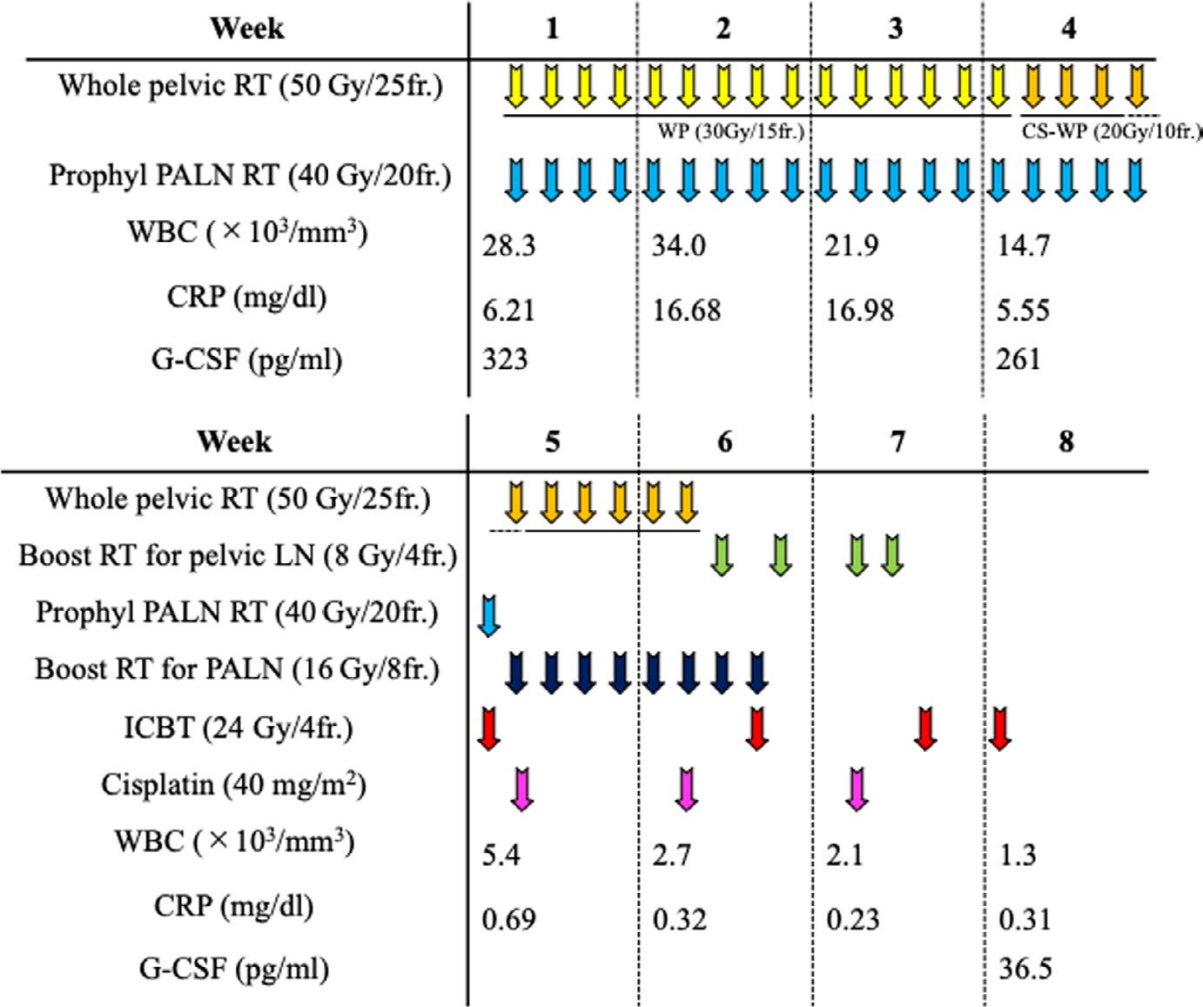
Schematic presentation of the treatment protocol. CS, central shielding; CRP, serum C‐reactive protein; fr, fractions; G‐CSF, serum granulocyte‐colony stimulating factor; ICBT, intracavitary brachytherapy; LN, lymph node; PALN, para‐aortic lymph node; Prophyl, prophylactic; RT, radiotherapy; WBC, white blood cell count in the peripheral blood; WP, whole pelvis

The patient received concurrent chemoradiotherapy using cisplatin. Radiotherapy consisted of external beam radiotherapy (EBRT) and intracavitary brachytherapy (ICBT). EBRT was administered as follows: (a) whole pelvic irradiation with 50 Gy in 25 fractions (the last 20 Gy was delivered using a central shielding technique); (b) prophylactic irradiation targeting PALN regions at a dose of 40 Gy in 20 fractions; and (c) boost irradiation to the enlarged lymph nodes (8 Gy in four fractions for pelvic lymph nodes and 16 Gy in eight fractions for PALNs). ICBT was delivered at a dose of 24 Gy in four fractions. Cisplatin (40 mg/m^2^) was administered weekly for a total of three cycles starting on day 29 after improvement of the fever and inflammatory response. The patient completed the radiotherapy regimen as planned (Figure 4). The overall treatment time was 49 days.

**FIGURE 5:**
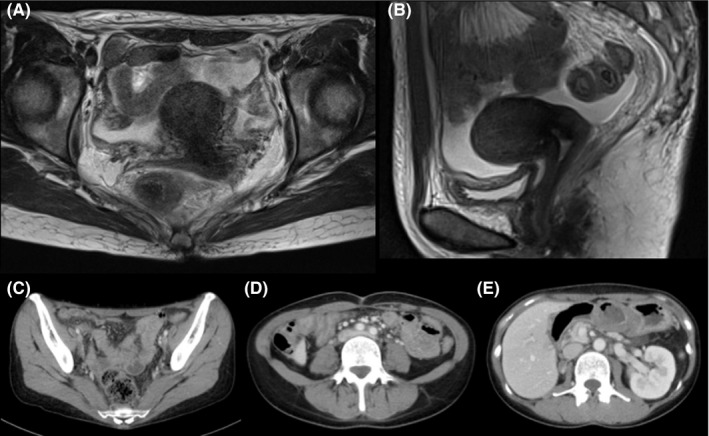
Magnetic resonance imaging (MRI) and computed tomography (CT) after completion of chemoradiotherapy. A, B, MRI of axial and sagittal planes, respectively, showing the uterine cervix. C‐E, CT image showing the external iliac, common iliac, and para‐aortic lymph node regions, respectively

After completion of the treatment, the tumor achieved almost complete response as assessed by the Response Evaluation Criteria in Solid Tumors (version 1.1) (Figure 5). Serum G‐CSF and SCC levels improved to within‐normal limits (36.5 pg/mL and 0.8 ng/mL, respectively). The patient experienced the following acute adverse effects as assessed by the Common Terminology Criteria for Adverse Effects (version 4.0): nausea (Grade 3), leukopenia (Grade 3), neutropenia (Grade 3), diarrhea (Grade 2), anemia (Grade 2), and thrombocytopenia (Grade 1). These adverse effects improved to Grade 0 within 2 months after completion of the treatment.

**FIGURE 6:**
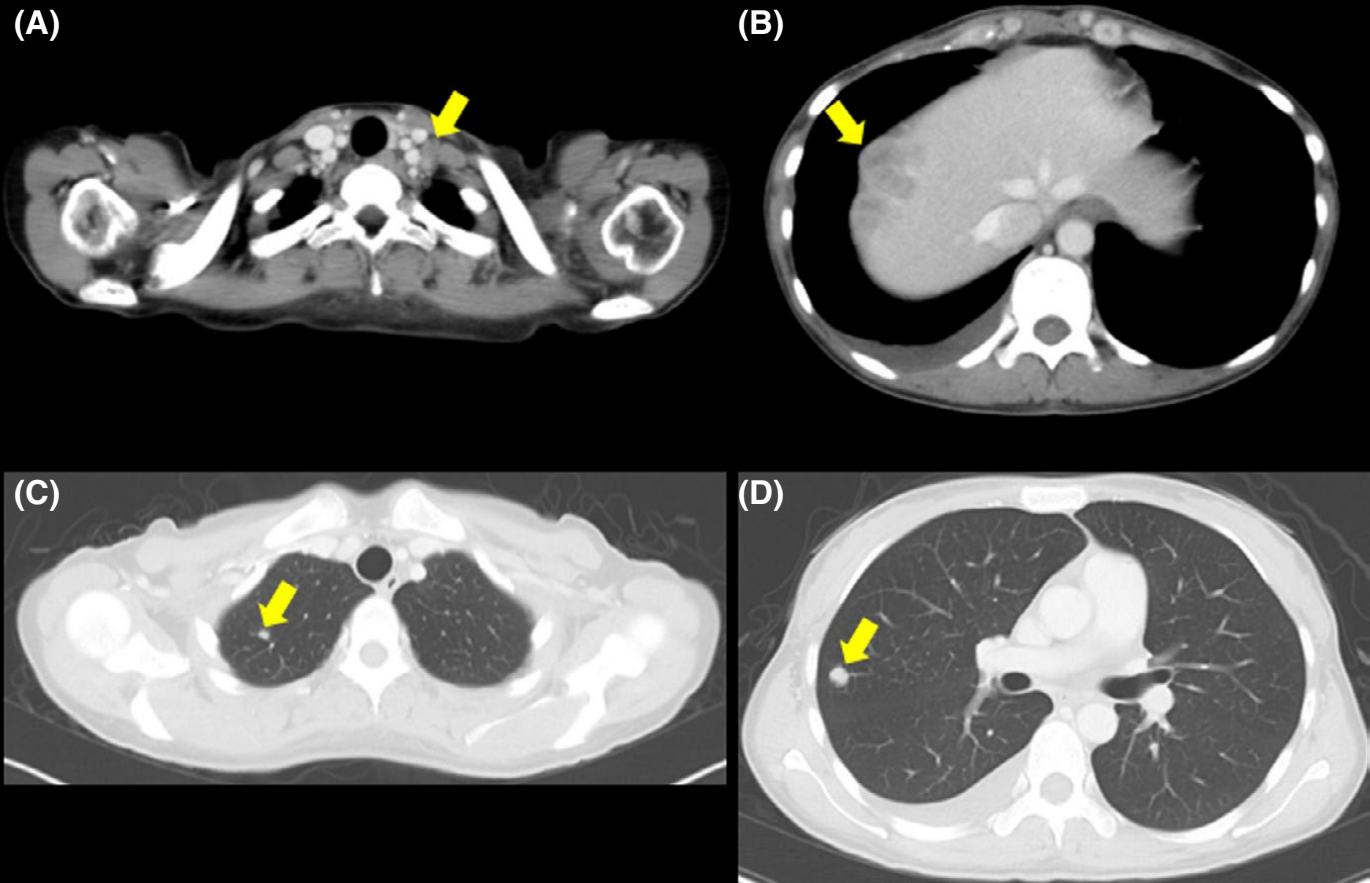
Computed tomography (CT) at 2 mo after completion of chemoradiotherapy. Metastatic tumors (arrows) in (A) the left supraclavicular lymph node, (B) the liver, and (C, D) the lung

**FIGURE 7:**
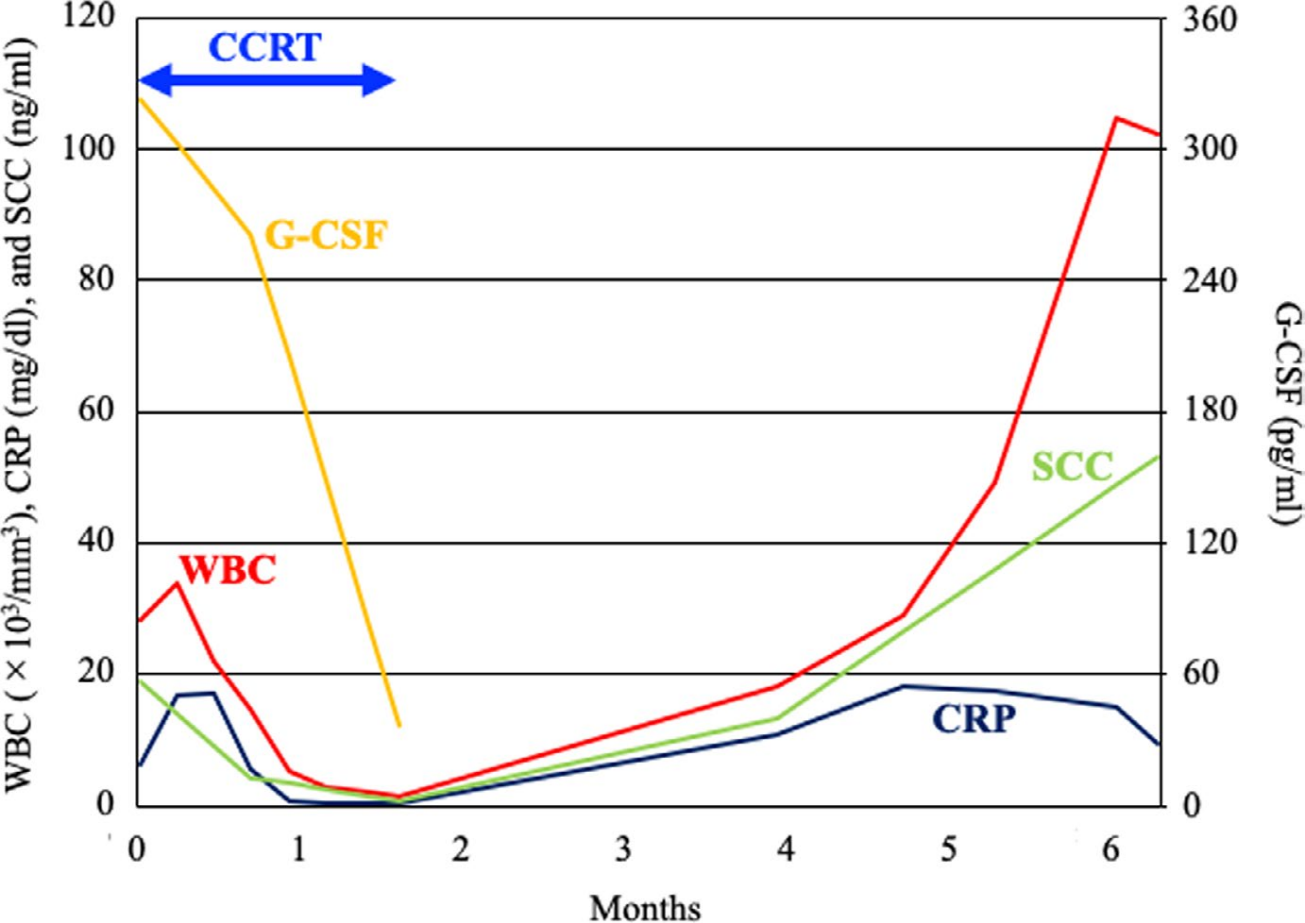
Kinetics of laboratory markers during the clinical course. CRP, serum C‐reactive protein; G‐CSF, serum granulocyte‐colony stimulating factor; SCC, serum squamous cell carcinoma antigen; and WBC, white blood cell count in the peripheral blood

The patient developed a fever (38.3°C) 2 months after completion of the treatment. CT examination showed multiple metastases to a left supraclavicular lymph node, the liver, and the lung (Figure 6). The patient was considered ineligible for chemotherapy because of the high inflammatory response (CRP, 18.30 mg/dL) and was provided with best supportive care. CT performed 4 months after completion of the treatment showed no evidence of infield recurrence. Serum G‐CSF was not assessed after the metastases; however, a remarkably high WBC count (104.8 × 10^3^/mm^3^) suggested that the G‐CSF‐producing property of the tumor was preserved at the metastatic sites (Figure 7). The patient expired from disease progression 5 months after completion of the treatment. Permission for an autopsy was not granted from the relatives at the time of mortality.

**FIGURE 8:**
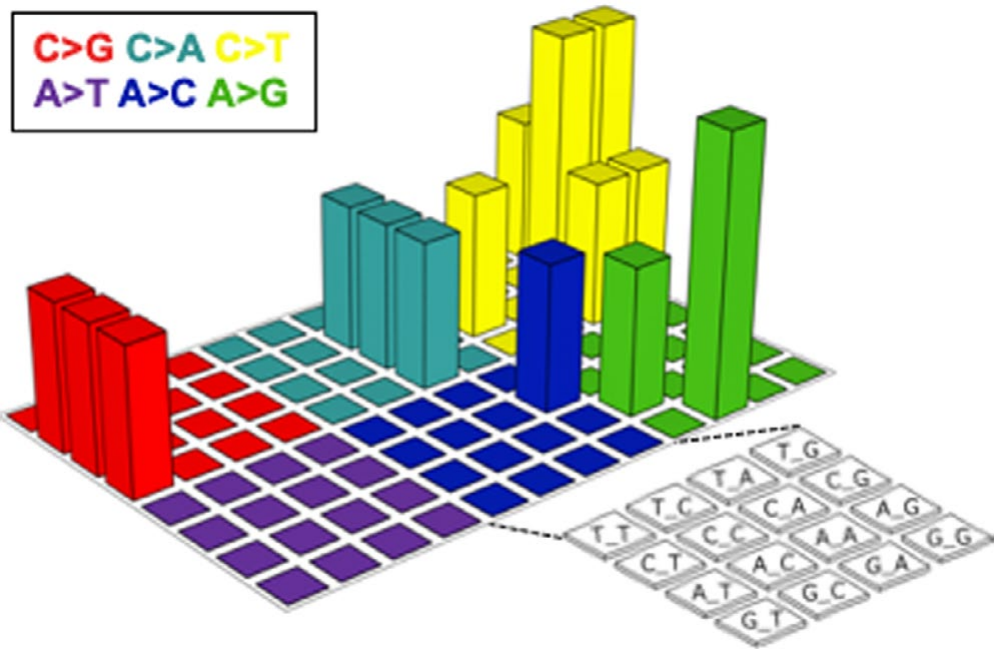
Mutation spectrum in a three‐base context^15^

To better understand the biological basis of this aggressive tumor, we analyzed mutation profiles. DNA was extracted from a pretreatment biopsy specimen in which the tumor cell content was higher than 50%. The exons of 409 cancer‐related genes (Table S1) were sequenced using the Ion AmpliSeq Comprehensive Cancer Panel (Thermo Fisher Scientific). After quality filtering, the number of sequencing reads per sample was 15.8 million, and the coverage depth was 945 reads per base (Table S2). Somatic mutations were identified using the analytical pipeline described previously.^14^ Sixteen nonsynonymous (Table 1) and four synonymous mutations were identified. The mutation spectrum in a three‐base context did not represent the APOBEC signature (ie, enrichment of substitution of a C preceded by a T into either T, G, or A) typical of HPV‐associated cancers^15^ (Figure 8). Of the 16 nonsynonymous mutations identified, *PIK3C2B* (T879N), *KDR* (V297I), and *TET2* (P29R) were registered in the Catalogue of Somatic Mutations in Cancer^16^ as recurrent mutations.

**Table 1:** Somatic nonsynonymous mutations identified in this study

Gene	Mutation	Mt count in COSMIC
*IGF2R*	L252V	0
	S1194L	0
	L2008V	0
*KDR*	V297I	16
*MTR*	N919G	0
*NOTCH4*	L13_L16del	0
*PIK3C2B*	T879N	2
*PLEKHG5*	I156T	0
*PTPRT*	D530A	0
*RNF213*	H5074T	0
*SAMD9*	V549L	0
*SYNE1*	A2795V	0
*TET1*	D162G	0
*TET2*	P29R	8
	V218M	0
*UBR5*	A1245S	0

Abbreviations: COSMIC, Catalogue Of Somatic Mutations In Cancer; MT, mutation.

## 3 DISCUSSION

Production of G‐CSF by cancer cells was first reported in 1977 by Asano et al^17^ In that report, the diagnostic criteria for G‐CSF‐producing cancer were as follows: (a) abnormally high WBC count in the peripheral blood with no evidence of infection; (b) abnormally high serum G‐CSF level; (c) decrease in the WBC count or G‐CSF level after cancer treatment; and (d) G‐CSF expression in tumor tissues. The authenticity of criterion *d* is controversial because studies indicate that the sensitivity for this criterion is not high (ie, approximately 70%).^18^, ^19^ Based on criteria a‐c, a literature search using PubMed with the search terms “*uterine cervical cancer*” AND “*granulocyte colony stimulating factor*”, followed by full‐manuscript examination, identified only 14 cases of G‐CSF‐producing UCC reported to date (

Table 1). The median age of patients was 64 years, and the histopathological type was squamous cell carcinoma in 64% of the cases. These patient demographics were in line with those reported for UCC in general.^20^, ^21^ Seventy‐five percent of the patients exhibited elevated serum CRP levels before the initial treatment. Seventy‐five percent of the patients experienced local recurrence or distant metastasis within 6 months after the initial treatment, suggesting resistance to conventional treatment and poor prognosis. The present case had a stage IIB tumor with PALN involvement and expired 5 months after completion of chemoradiotherapy because of multiple distant metastases. Previous studies show that the 5‐year overall survival rate of cohorts predominantly composed of PALN‐positive stage IIB tumors treated with chemoradiotherapy is approximately 40%‐60%,^22^, ^23^ highlighting the aggressive behavior of the tumor in the present case.

**Table 2:** Summary of reports on G‐CSF‐producing UCC.

Age	Stage	Histology	G‐CSF (pg/mL)	WBC (×10^3^/µL)	CRP (mg/dL)	Initial Tx	PFS (M)	Rec site	Rec Tx	OS (M)	Reference
56	IB2	Sq	197	12.9	7.0	Surgery	0.5	Parametrium	BSC	2	6
39	IB2	Sq	50	13.9	1.8	Surgery‐RT	0.25	Brain, skin, lung	CT	5	7
72	IB2	Sq	248	20.6	4.8	CCRT	6	Uterus. lung	S‐CT	15	7
64	IIA2	Cs	1500	48.0	NA	RT	0.25	Skin, bone	RT	2	8
64	IIB	Ad	148	11.8	3.8	RT	0.25	SCLN, liver,lung	BSC	3	9
43	IIB	Sq	323	28.3	6.2	CCRT	2	SCLN, liver,lung	BSC	5	Present case
58	IIB	Sq	58	22.5	6.6	CCRT	NA	NA	NA	24	10
75	IIB	NA	223	31.7	2.6	CIRT	‐	‐	‐	108+	11
41	IIIB	Sq	106	34.4	1.5	CCRT	3	Liver	CT	9	7
76	IIIB	Sq	642	31.1	5.4	CIRT	‐	‐	‐	30+	11
71	IIIB	Sq	195	30.0	NA	RT	‐	‐	‐	8+	12
59	IVB	Sq	875	13.7	0.6	RT‐AC	1	Lung, LN	BSC	3	7
70	IVB	Sm	269	17.1	0.8	CT‐RT‐CT	0	Liver	BSC	11	13

Note

Laboratory test results before initial treatment are shown.

Abbreviations: +, still alive; Ad, adenocarcinoma; BSC, best supportive care; CCRT, concurrent chemo‐radiotherapy; CIRT, carbon ion radiotherapy; CRP, serum C‐reactive protein before initial treatment; Cs, carcinosarcoma; CT, chemotherapy; G‐CSF, granulocyte‐colony stimulating factor; LN, lymph node; M, months; NA, not available; OS, overall survival; PFS, progression‐free survival; Rec, recurrence; RT, radiotherapy; SC, supraclavicular; Sm, small cell carcinoma; Sq, squamous cell carcinoma; Tx, treatment; UCC, uterine cervical cancer; WBC, white blood cell count before initial treatment.

The mutational landscape of G‐CSF‐producing cancer remains unclear. A small number of studies have analyzed a few genes of interest in G‐CSF‐producing lung cancers. Preclinical studies identified inactivating mutations in *TP53* and *RASSF1A*,^24^ and reported conflicting results on the presence of activating mutations in *KRAS*,^24^, ^25^ whereas activating mutations in *EGFR* were identified in a clinical tumor.^26^ To the best of our knowledge, this is the first study reporting the mutation profiles of G‐CSF‐producing cancer using a clinically available sequencing panel containing hundreds of genes. In the present case, the mutation spectrum in a three‐base context was not typical for UCC.^15^ None of the mutations in *PIK3C2B*, *KDR*, and *TET2* identified in the present case were reported in two previous large‐scale landmark studies of UCC‐mutation profiles.^27^, ^28^ By contrast, mutations in genes such as *PIK3CA*, *PTEN*, *STK11*, *KRAS*, *ARID1A*, *EP300*, and *FBXW7*, which were recurrently identified in previous landmark studies, were absent in the present case. Taken together, these data suggest that G‐CSF‐producing UCC is genetically distinct from other UCCs. Further compilation of clinical sequencing data is needed to elucidate the mutational landscape of G‐CSF‐producing cancer.

G‐CSF‐producing UCCs show resistance to conventional treatment (Table 2). Therefore, treatment strategies specific for this type of cancer need to be established. One candidate strategy is the use of molecular‐targeted drugs selected based on actionable mutation profiles. *PIK3C2B* and *KDR* encode class II phosphoinositide‐3‐kinase isoform C2β and vascular endothelial growth factor receptor 2, respectively. Therefore, cases such as the present case, which harbor somatic mutations in these genes, could be treated with inhibitors of the relevant PI3K/AKT/mTOR pathway.^29^, ^30^ Another candidate strategy is carbon ion radiotherapy. Patients with stages IIB and IIIB disease treated with carbon ion radiotherapy survived 108 and 30 months, respectively, with no evidence of recurrence.^11^ The efficacy of the precision medicine approach and that of carbon ion radiotherapy for G‐CSF‐producing UCCs should be further investigated.

As a limitation of this study, we were unable to assess the expression of G‐CSF in the tumor tissue using immunohistochemistry because of insufficient sample material.

## 4 CONCLUSIONS

We reported a case of G‐CSF‐producing UCC that showed rapid disease progression after definitive chemoradiotherapy leading to an overall survival period of 5 months. The aggressive behavior of this tumor against conventional treatment was in line with the findings described in previous reports on G‐CSF‐producing UCCs. This study is the first report describing the somatic mutation profile of G‐CSF‐producing UCC. The data presented will expand our biological understanding of this cancer subset, which warrants further investigation.

## CONFLICT OF INTEREST

None declared.

## AUTHOR CONTRIBUTIONS

SS and KA: treated the patient and drafted the manuscript; YY: analyzed sequencing data; YT: performed pathological review; T. Oike: analyzed sequencing data and finalized the manuscript; T. Ohno: supervised the study.

## ETHICAL APPROVAL

The enrollment of the patient was approved by the Institutional Ethical Review Committee of Gunma University Hospital (approval number: 1109). The Institutional Ethical Review Committee waived the requirement for written informed consent from the patient because of the retrospective and noninvasive design of the study. The study was conducted in accordance with the ethical principles of the Declaration of Helsinki.

## Supporting information

Tab S1

Tab S2
